# Posterior Corneal Asphericity Effect on Postoperative Astigmatism after EDOF Intraocular Lens Implantation in Cataract Patients

**DOI:** 10.1155/2021/1877516

**Published:** 2021-11-03

**Authors:** Mark Rabinovich, Ivo Guber, Laëtitia Jessy Niegowski, Ana Maria Aramburu del Boz, Danial Al Khatib, Jean-Pascal Genestier, Jerome Bovet

**Affiliations:** ^1^OnO, Ophthalmology Network Organisation, Clinique de L'Oeil SA, Onex, Geneva, Switzerland; ^2^University Hospital of Geneva, Department of Opthalmology, Geneva, Switzerland

## Abstract

**Aim:**

To assess the impact of posterior corneal asphericity on postoperative astigmatism.

**Methods:**

We included retrospectively 70 eyes of 70 patients that underwent cataract surgery. We included data of the *Q* value, *K*_max_, *K*1, *K*2, astigmatism AL, and ACD. We performed a vectorial analysis to calculate the astigmatic vectors.

**Results:**

Seventy eyes were evaluated. 40 eyes were of females (58%) and 30 of males (42%). The average cohort age was 73 ± 8.9 years. Axial length (AL) was 23.5 ± 0.9, anterior chamber depth (ACD) was 3.13 ± 0.3, and the average posterior *Q* value was −0.35 ± 0.2. The only significant predictive variable for the correction index (CI) was the posterior *Q* value (*r* = 0.24, *p* < 0.05) and for the surgically induced astigmatism (SIA) (*β* = 0.34, *r* = 0.58, *p* < 0.05).

**Conclusion:**

Posterior corneal surface asphericity significantly influences the surgically induced astigmatism and the overcorrection for cataract patients after Lucidis EDOF IOL implantation.

## 1. Introduction

The *Q* value serves as a key parameter reflecting the corneal asphericity and optical properties including power of refraction, spherical aberration, and aberration variability.

A negative *Q* value denotes a prolate structure of the cornea, while a positive value is consistent with an oblate form [1]. Both anterior and posterior corneal asphericities do not have any correlation to each other, with the latter presenting a more aspherical shift with age [2].

Various measurement methods such as Scheimpflug imaging (Pentacam®, Oculus Optikgeräte GmbH, Germany) can be used to measure the *Q* value.

Corneal asphericity may be a possible source of errors in power calculations. This implies that (intraocular lens) IOL power calculation can be refined by taking asphericity into account and including measurements of both corneal surfaces [3]. Consideration of the asphericity of the cornea is crucial to minimize postoperative refractive surprises. In the past, the posterior asphericity of the cornea has received less or almost no attention in the analysis of potential factors leading to IOL refraction calculation errors.

Measurement derived the errors of different types; it was previously found that the measurement methods of the cornea for IOL power calculation do not differ significantly [4]. Although, the smallest mean absolute error (MAE) was established using the Scheimpflug method [4].

Savini et al. showed an influence of the anterior asphericity on the refractive results [5]. Until recent introduction of anterior segment optical coherence tomography (AS-OCT), there has been a lack of evaluation of the posterior asphericity in IOL power calculation.

The purpose of the study is to evaluate the impact of posterior corneal asphericity on postoperative astigmatism after Lucidis EDOF IOL implantation in cataract patients.

## 2. Methods

It is a one-center, retrospective study of 70 eyes of 70 patients following cataract surgery with EDOF IOL implant (Lucidis EDOF, Swiss Advanced Vision, SAV-IOL SA, Neuchâtel, Switzerland). Surgeries were performed by 4 experienced surgeons between 2018 and 2020. All patients gave their consents before the operation.

Exclusion criteria: diabetic retinopathy, glaucoma, uveitis, macular degeneration, corneal opacifications, or astigmatism of >1.00D, and age <18 years.

### 2.1. Intraocular Lens

The Lucidis single-piece foldable refractive/aspheric IOL has a 360° square edge design with closed loop haptics, 6.0 mm optical diameter, and a total diameter of 10.8 mm/12.4 mm. It is composed from hydrophilic acrylic with a 26% water content. Optically, Lucidis IOL has a 1 mm aspheric zone that occupies the center of the IOL, the axicon, and is surrounded by a 6 mm refractive ring.

### 2.2. Surgery

Every surgery was performed under local anesthesia and a venous sedation with Rapifen and propofol on demand. All interventions were performed using the same standard protocol on a temporal side, 2 mm main incision.

### 2.3. Posterior *Q* Value

Posterior asphericity *Q* value was included from the data of the rotating Scheimpflug camera, which is known to have the lowest MAE [4]. All values were obtained at a diameter of 8.0 mm at 25°. A prolate cornea (−1 < *Q* < 0) was with a steeper central curvature compared to the peripheral curvature, and an oblate cornea was (*Q* > 0), with a flatter central curvature compared to the peripheral curvature.

### 2.4. Lens Power Calculation

SRK-T formula was used for IOL power calculation. Axial length, anterior chamber depth measurements, and the target cylinder and axis were found using IOL Master 500 (Zeiss).

### 2.5. Vectorial Analysis

Initially, all the negative cylinders (i.e., −ve) values were converted to positive cylinders (i.e., +ve) by turning the axis by ninety degrees and by taking the absolute value of the magnitude. The target-induced astigmatism vector (TIA) and surgically induced astigmatism vector (SIA) vectors were then calculated from the preoperative and postoperative astigmatism values using the ASSORT® vector calculator according to method of Alpins et al. [6].

The magnitude of error as well as the angle of error (ME and AE) were calculated as the arithmetic difference between the magnitudes of SIA and TIA and the angle of the vectors of SIA and TIA, respectively.

Likewise, the correlation index (CI) was the ratio of SIA to TIA.

### 2.6. Statistical Analysis

Quantitative and qualitative variables were described in terms of size, mean, median, and standard deviation, as well as of range.

Data analysis was performed using Excel (Microsoft, WA, USA). The analyses were the independent *t*-test for two means and the Wilcoxon test for nonparametric continuous data. Results were considered statistically significant if *p* < 0.05.

We used SPSS version 26 (IBM, USA) for the ANOVA test and multivariant regression analysis. Results were considered statistically significant if *p* < 0.05.

After the analysis, multivariate models were created to adjust for variables that could be potential confounders, which included all variables with *p* < 0.10 in the univariate regression model.

## 3. Results

We included 70 eyes of 70 patients, with 1-month postoperative data: 30 eyes of males (42%) and 40 eyes of females (58%). The average age of was 73 ± 8.9 years.

The mean axial length (AL) is 23.5 ± 0.9 mm, the mean anterior chamber depth (ACD) is 3.13 ± 0.3 mm, and the mean posterior *Q* value is −0.35 ± 0.2, as given in Table 1.

Two separate univariate analyses were performed for the corneal parameters *K*_max_, *K*1, *K*2, astigmatism, and anterior and posterior asphericity for the dependent variables SIA and CI, as given in Table 2. After identification of possible confounders (a variable with *P* < 0.1), we performed a multivariate analysis, as given in Table 3.

For SIA as the dependent value, posterior *Q* value adjusted for confounders had a significant effect (*β* = 0.34, *r* = 0.58, *p* < 0.05).

The univariate analysis for CI yielded no variables with *p* > 0.1, and the only significant predictive variable for CI was the posterior *Q* value (*r* = 0.24, *p* < 0.05).

**Figure 1:**
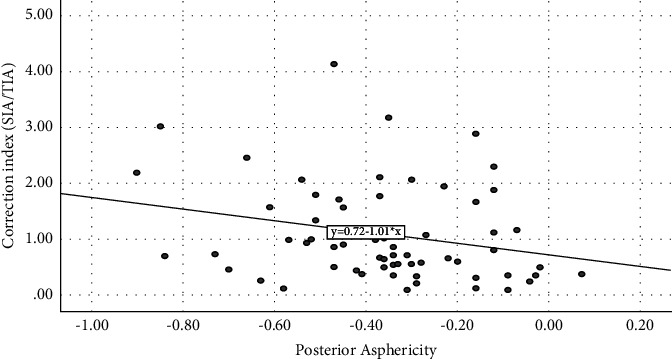
The correlation graph between the posterior *Q* value and the correlation index.

Figure 1 shows the correlation between those parameters. The CI reflects the amount of correction. It is bigger than 1 if there is overcorrection and less than 1 if there is an undercorrection. Thus, the more prolate the posterior cornea (expressed by a more negative posterior *Q* value), the more positive is the CI value. According to Figure 1, for posterior *Q* values more negative than −0.3 (more prolate), astigmatism was overcorrected.

Further regression analyses did not yield significant results for the variables of magnitude and angle of error (ME and AE).

**Figure 2:**
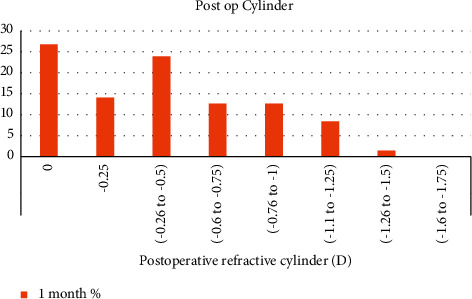
Postoperative refractive cylinder.

We had 65% of the eyes with a resulting postoperative cylinder of less than 0.5D at 1-month postoperatively, as shown in figure 2.

**Figure 3:**
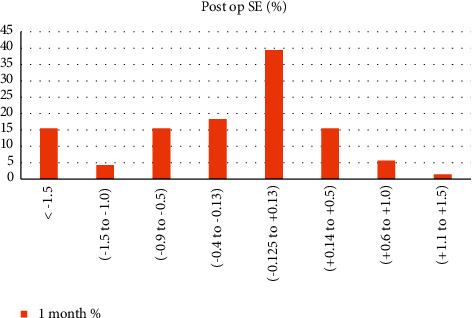
Postoperative spherical equivalence.

Seventy-three percent of the eyes had a 1-month postoperative spherical equivalent between −0.4 and 0.5D, as shown in figure 3.

## 4. Discussion

We demonstrated, in this study, a significant influence of that posterior corneal asphericity on the surgically induced astigmatism as well as the correction index for cataract patients after Lucidis EDOF IOL implantation.

Cataract surgeons always aim for a better correction of astigmatism in order to achieve a satisfactory visual acuity in the operated eyes. It is recommended to aim for astigmatism of less than 0.5D in order to prevent postoperative visual acuity degradation [7]. Manifest astigmatism can be lowered by paying attention to both corneal surfaces. Methods that take into account the posterior corneal astigmatism are advocated for that purpose [8].

The negative power of the posterior corneal aspect was demonstrated to cause the cornea to behave oppositely to that of the anterior corneal aspect. Thus, the steeper the curve of a certain posterior corneal meridian, the more negative the power [8]. Eventually, producing an against-the-rule (ATR) refractive astigmatism as the corneal meridian is steeper. The latter is correlated to PCA, whose mean values were taken into consideration by Koch et al. for TCA prediction. They aimed to leave the eyes after a cataract surgery with small amounts of with-the-rule (WTR) refractive astigmatism [9]. They concluded that IOL selection on the basis of anterior corneal measurements could cause overcorrection in the eyes that have WTR and undercorrection in the eyes that have ATR.

In our study, we found that in a more prolate posterior cornea, it is to say a more negative posterior asphericity is correlated to a more overcorrection of astigmatism, expressed by the correction index >1.0.

Previously, it has been argued that astigmatic overcorrection is of benefit for the result of astigmatism and is more likely to provide with spectacle independence. In addition, given the inclination of anterior corneal astigmatism to shift from with-the-rule (WTR) to against-the-rule (ATR) with age, aiming on residual WTR astigmatism could be of benefit for patients over the long term [9, 10].

Regarding the surgically induced astigmatism (SIA), it is argued that there is a resulting flattening effect along the orientation of the incision. If the incision is made oblique to the steepest corneal meridian, it results in a torsional effect on the preexisting astigmatism [6, 11, 12].

On the one hand, it is assumed that SIA measured upon keratometry data is free from error. However, on the other hand, most studies report a minute level of error in the repeatability of readings especially when describing astigmatism, which are significant when compared to the low levels of SIA reported [13–16].

If we locate the sources of error, we can minimize their influence on SIA and its use in the future.

We found a significant correlation between the posterior corneal asphericity and the SIA (*β* = 0.34, *r* = 0.58, *p* < 0.05). Thus, the more prolate the cornea, expressed by a more negative posterior asphericity, the higher is the surgically induced astigmatism. Since the correlation is adjusted for confounding factors, we can state that the posterior asphericity could be a source of SIA change that could be taken in consideration preoperatively.

As far as we know, the impact of posterior corneal asphericity those parameters have rarely been investigated.

The limitations of this study are its retrospective nature; likewise, the rather small number of the eyes is evaluated.

Furthermore, a bias is caused when including several surgeons and refractions performed by numerous ophthalmologists, despite it being a single-center study.

In addition, the extrapolation of the posterior corneal power by IOL Master 500 is when normally measuring central corneal power on the anterior corneal surface, as a result of a fixed ratio assumed between the anterior and posterior curvatures and a fixed refractive index.

To conclude, the posterior corneal surface asphericity significantly affects surgically induced astigmatism as well as the correction index for cataract patients after Lucidis EDOF IOL implantation.

## Figures and Tables

**Table 1:** Demography of population.

	*N* = 70
Age	72.7 ± 8.9
Sex (male/female)	30/40
Lucidis IOL power	19.6 ± 3.47
*K*_max_	44.8 ± 1.5
*K*1	43.2 ± 1.3
*K*2	43.9 ± 1.5
Astigmatism	0.76 ± 0.57
Anterior *Q* value	−0.24 ± 0.18
Posterior *Q* value	−0.35 ± 0.2
ACD	3.13 ± 0.3
Axial length	23.5 ± 0.98
Target-induced astigmatism (TIA)	0.75 ± 0.54
TIA axis	82 ± 62.3
Surgically induced astigmatism	2.75 ± 17
SIA axis	89 ± 74.5
Magnitude of error	2 ± 16.9
Angle of error	7.3 ± 87.7
Correction index (CI)	2.37 ± 10.8

**Table 2:** Univariate analysis for corneal parameters for SIA and CI.

Univariate analysis, *n* = 70				Univariate analysis, *n* = 70			
Dependent value, SIA				Dependent value, CI			
	B	SD	*P* value		B	SD	*P* value
*K*_max_	−0.09	0.04	0.016	*K*_max_	0.08	0.08	0.28
*K*1	0.04	0.5	0.45	*K*1	0.03	0.08	0.77
*K*2	−0.08	0.44	0.07	*K*2	0.01	0.8	0.89
Astigmatism	0.55	0.13	9*E* − 06	Astigmatism	−0.13	0.28	0.65
Anterior *Q*	−0.18	0.29	0.53	Anterior *Q*	0.45	0.55	0.4
Posterior *Q*	−0.63	0.26	0.02	Posterior *Q*	−1.01	0.49	0.045

**Table 3:** Multivariate analysis of possible confounding corneal parameters for SIA.

Multivariate analysis				
Dependent value, SIA				
	Beta-coefficient	*P* value	*R*	*R* ^2^ (%)
*K*_max_	0.25	0.27	0.58	34
Astigmatism	0.37	0.01		
Posterior *Q*	−0.34	0.02		

## Data Availability

The data used to support the findings of this study are available from the corresponding author upon request.
